# Blastomycosis presenting as an isolated progressive painless verrucous skin lesion

**DOI:** 10.1002/ccr3.2556

**Published:** 2020-02-25

**Authors:** Saira Butt

**Affiliations:** ^1^ Division of Infectious Diseases Indiana University School of Medicine Indianapolis IN USA

## Abstract

A painless progressive verrucous skin plaque in an endemic area should raise suspicion for blastomycosis resulting in prompt biopsy with fungal stains and culture. Skin is a common extrapulmonary site. Itraconazole is treatment of choice.

## Graphical abstract

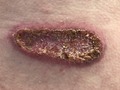

A 57‐year‐old Caucasian man from Indiana was referred to infectious diseases clinic for a skin lesion. A nonpruritic and painless skin lesion had been present on his lower back for a year and had progressively enlarged. Topical steroids worsened the lesion. Patient was asymptomatic. Physical examination showed a 10 × 3 cm raised erythematous verrucous plaque with stuck‐on appearance with dry scaly raised brown borders.^1^ (Figure 1) The patient worked as a car mechanic, lived in a suburban old house, denied any travel, was monogamous with his wife, and had no adventurous hobbies. His labs and chest X‐ray were normal. The lesion was biopsied and showed thick‐walled fungal spores in the dermis with broad‐based budding yeast consistent with blastomycosis.^2^ Patient's urine and serum blastomyces antigens were negative. Oral itraconazole 200 mg twice a day was initiated. Itraconazole was tolerated well, and patient had adequate drug level. On 3‐month follow‐up, the lesion had become flat and less scaly. (Figure 2) On 6‐month follow‐up, it had become smooth and faded. (Figure 3) Itraconazole was stopped at 6 months, and patient continued to do well clinically without any new symptoms or lesions.

**Figure 1:**
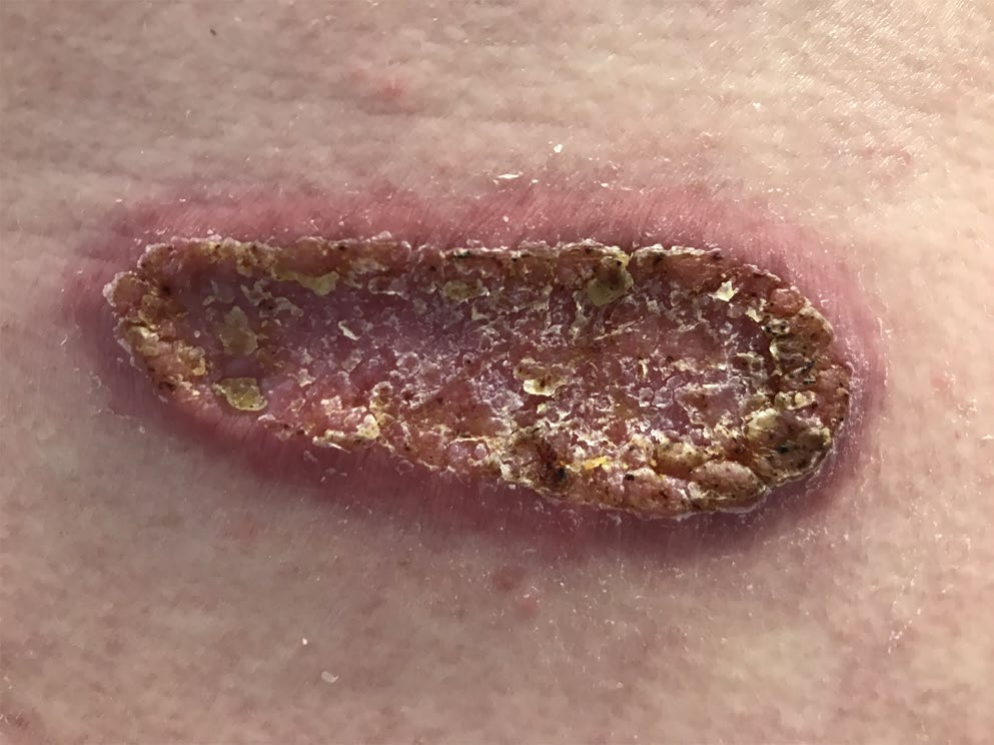
Initial presentation: painless progressive verrucous skin lesion

**Figure 2:**
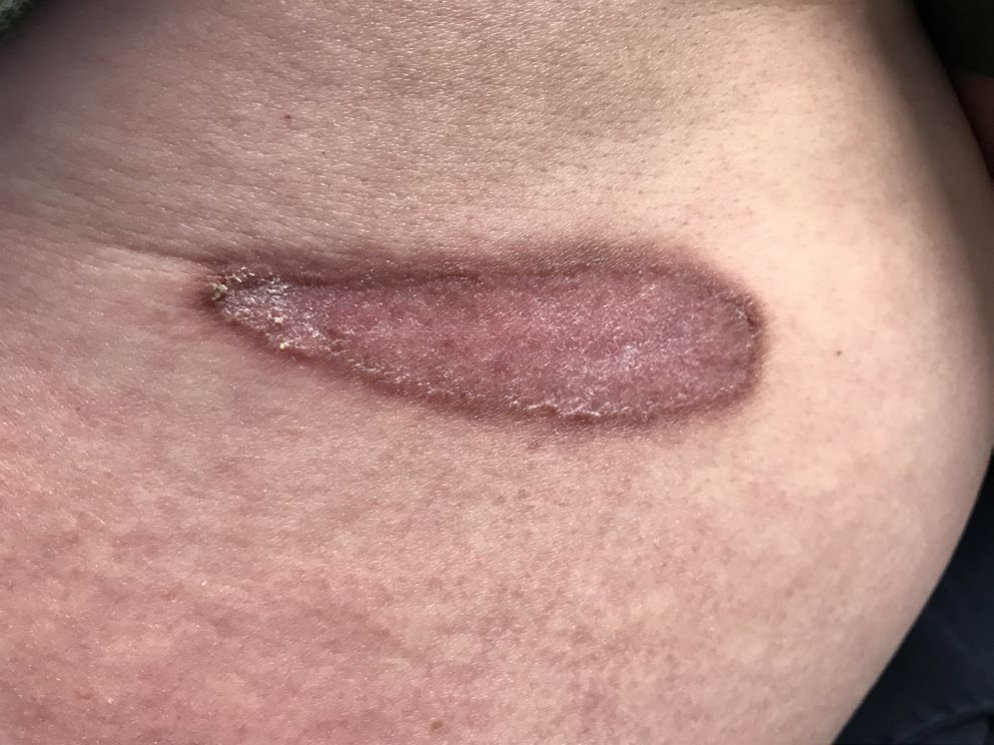
Three‐month follow‐up: flat and less scaly skin lesion

**Figure 3:**
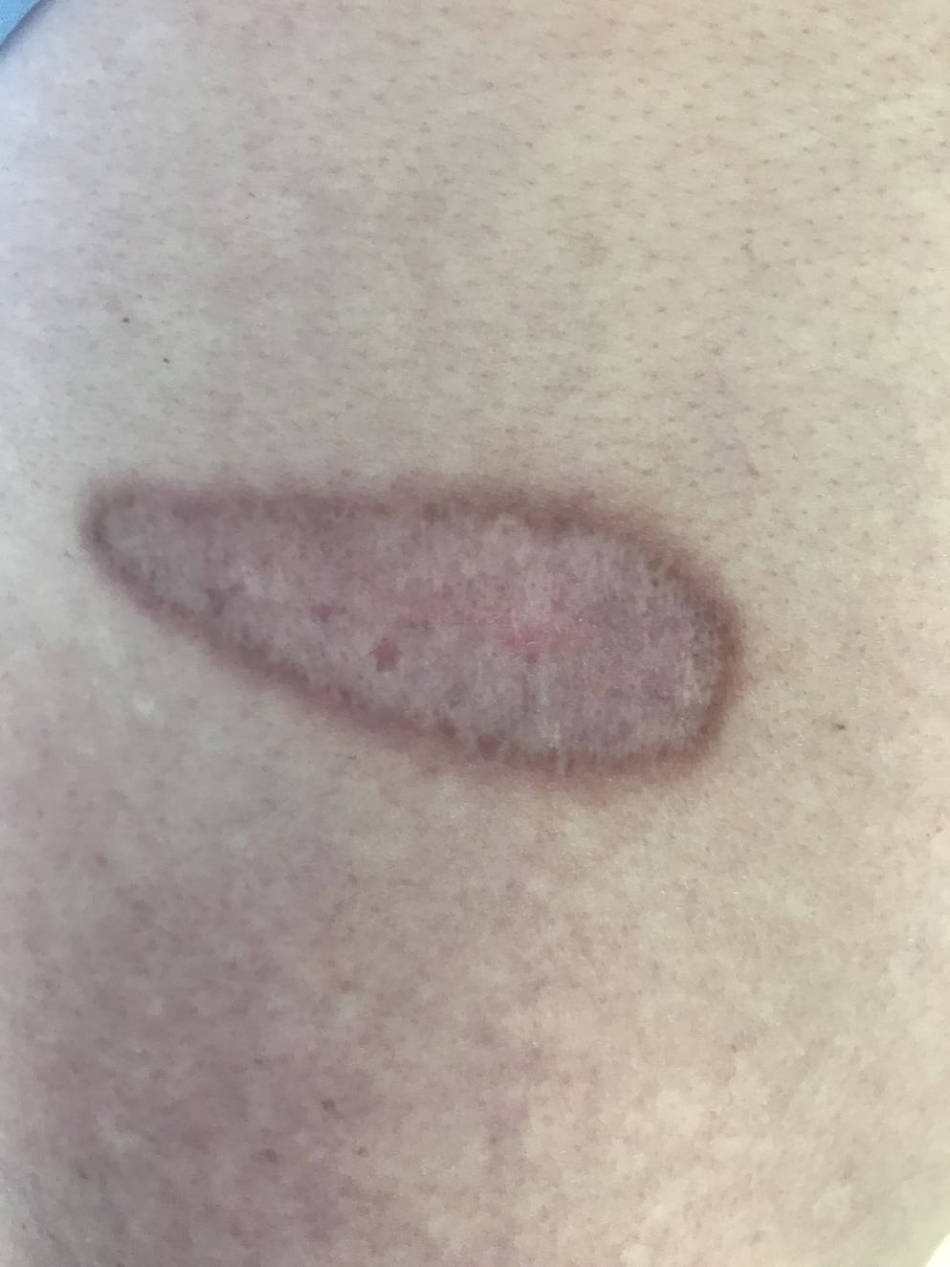
Six‐month follow‐up: smooth and faded skin lesion

## CONFLICT OF INTEREST

None declared.

## AUTHOR CONTRIBUTIONS

SB: drafted and reviewed the article.
